# Blocking Orbital π‐Conjugation to Boost Spin‐Orbit Coupling in Carbonyl‐Embedded Polycyclic Heteroaromatic Emitters

**DOI:** 10.1002/anie.202503371

**Published:** 2025-03-22

**Authors:** Rajat Walia, Xiaochun Fan, Le Mei, Weixiong Guo, Kai Wang, Chihaya Adachi, Xian‐Kai Chen, Xiao‐Hong Zhang

**Affiliations:** ^1^ Institute of Functional Nano and Soft Materials (FUNSOM) Joint International Research Laboratory of Carbon‐Based Functional Materials and Devices Soochow University Suzhou Jiangsu P.R. China; ^2^ Center for Organic Photonics and Electronics Research (OPERA) Kyushu University 15 Fukuoka 819‐0395 Japan; ^3^ Department of Chemistry City University of Hong Kong Kowloon Hong Kong SAR P.R. China; ^4^ Jiangsu Key Laboratory for Carbon‐Based Functional Materials & Devices Soochow University Suzhou Jiangsu P.R. China; ^5^ Jiangsu Key Laboratory of Advanced Negative Carbon Technologies Soochow University Suzhou Jiangsu P.R. China

**Keywords:** Multi‐resonance emitters, Spin‐orbit coupling, Thermally activated delayed fluorescence

## Abstract

Both reducing singlet‐triplet energy gaps (Δ*E*_S1T1_) and enhancing spin‐orbit couplings (SOCs) are key to improving reverse intersystem crossing rates (*k*_RISC_) in thermally activated delayed fluorescence (TADF) materials. While considerable efforts have focused on reducing Δ*E*_S1T1_, investigations on SOCs remain limited. Here, blocking π‐conjugation in carbonyl‐embedded polycyclic heteroaromatic (PHA) molecules as potential approach to elevate ππ* excitation energy, allowing its hybridization with nπ* excitation, thereby increasing SOCs is proposed. Two proof‐of‐concept isomers, DNDK‐1 and DNDK‐2 are synthesized, with different orientations of carbonyl units. DNDK‐1 adopts a heavily twisted structure that hinders π‐conjugation, while DNDK‐2 remains quasi‐planar, maintaining stronger π‐conjugation. Experimental measurements reveals stark differences in their photophysical properties, with DNDK‐1 exhibiting faster *k*_RISC_ and much higher electroluminescence efficiency. The *ab‐initio* calculations elucidate that hindered conjugation in DNDK‐1 elevates ππ* excitation energy, enabling nπ*‐ππ* mixing, thus significantly boosting SOCs. In contrast, smooth π‐conjugation in DNDK‐2 leads to marginal nπ*‐ππ* mixing. In addition, utilizing groups composed of meta‐arranged carbonyl‐Ar‐carbonyl and meta‐arranged N‐Ar‐N units emerges as another approach to block π‐conjugation and enhance SOCs. This joint experimental and theoretical work provides promising pathways to enhance SOCs by blocking π‐conjugation, offering crucial insights for designing carbonyl‐embedded PHA emitters with larger SOCs.

## Graphical abstract

An effective approach, i.e., blocking orbital π‐conjugation via either molecular‐structure distortion or meta‐arrangement of chemical substituents is proposed, to boost spin‐orbit couplings (SOC) in carbonyl‐embedded polycyclic heteroatomic emitters. This work not only provides a deep insight into the structure‐property relationship on SOC but also opens the door for designing more efficient OLED emitters with large SOCs.

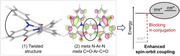

## Introduction

Polycyclic HeteroAromatic (PHA) molecular materials with the so‐called multi‐resonance (MR) effect have garnered significant interest due to their great potential for application in ultrahigh‐definition organic light‐emitting diode (OLED) displays and organic lasers. The rigidly π‐fused frameworks in these molecules substantially suppress the geometrical relaxation and thus offer unique advantages, such as narrow‐band emission with full‐width half maximum (FWHM) values of <20 nm.^[^ ^1^, ^2^, ^3^, ^4^, ^5^, ^6^, ^7^, ^8^, ^9^, ^10^ ^]^ However, OLEDs based on such materials suffer from severe efficiency roll‐off and poor device operational stability.^[^ ^4^, ^7^, ^11^ ^]^ One of the primary reasons is the slow rate of reverse intersystem crossing (*k*_RISC_) from triplet to singlet. Therefore, it is a pressing task to comprehend the factors responsible for elevating *k*_RISC_, in order to eventually improve the efficiency and stability of OLEDs based on PHA emitters with thermally activated delayed fluorescence (TADF).

For the feasibility of the RISC process, it is anticipated from the Fermi golden rule that the lowest singlet (S_1_) and triplet (T_1_) excited states should be proximal in energy to maintain narrow singlet‐triplet energy gaps (Δ*E*_S1T1_). In addition, there must be significant spin‐orbit coupling (SOC) between these two states to facilitate efficient *k*_RISC_.^[^ ^12^, ^13^ ^]^ Therefore, small Δ*E*_S1T1_ and large SOC both remain the core parameters for accelerating the RISC process. In recent years, a lot of attention has been paid to investigating factors impacting Δ*E*_S1T1_ in PHA‐based TADF materials.^[^ ^14^, ^15^, ^16^ ^]^ As a result, a significant number of PHA emitters have been developed with boron(B)‐nitrogen(N),^[^ ^1^, ^2^, ^17^, ^18^, ^19^ ^]^ boron(B)‐oxygen(O),^[^ ^20^, ^21^ ^]^ or carbonyl(C═O)‐nitrogen(N)^[^ ^22^, ^23^, ^24^, ^25^, ^26^, ^27^ ^]^ frameworks. In MR frameworks, the electron‐deficient (B or C═O) and electron‐rich (N) units are in the opposite resonance sites to separate spatial distributions of the highest occupied molecular orbital (HOMO) and the lowest unoccupied molecular orbital (LUMO), thus reducing the exchange energy (*K*_HL_) and Δ*E*_S1T1_ (since Δ*E*_S1T1_ is positively proportional to 2*K*_HL_)_._^[^ ^28^ ^]^ Although, Δ*E*_S1T1_ in these reported PHA emitters can be smaller than 0.1 eV in the mainstream BN‐based PHA emitters,^[^ ^7^ ^]^ their SOCs still remain unsatisfactory and are commonly negligible (≈0.1 cm*^−^* ^1^), which severely impedes RISC processes in such emitters.

To enhance SOCs in PHA‐based TADF emitters, the heavy‐atom effect of Selenium was incorporated, which eventually leads to a large SOC (≈2.8 cm^−1^) and a fast *k*_RISC_ (≈10^6^ sec^−1^). It demonstrated a promising maximum external quantum efficiency (EQE_max_) of ≈36.8% in an OLED with a binary emissive layer composed of a bipolar TADF host and a Se‐integrated MR emitter.^[^ ^29^ ^]^ However, OLEDs exploiting the Se‐integrated MR emitters show short device lifetimes, e.g., LT80 (the lifetime attenuating to 80% of the initial luminance) at an initial brightness of 1000 cd m^−2^ ≈1 h, which could be ascribed to the high chemical activity of organic selenides. Therefore, the design and development of PHA‐based TADF emitters composed of only Period‐2 elements with fast *k*_RISC_ are critical.^[^ ^29^, ^30^, ^31^ ^]^

Recently, a few carbonyl‐embedded PHA emitters have been reported, which not only achieved excellent Δ*E*_S1T1_ but some of them demonstrated remarkable SOCs, with the corresponding OLEDs achieving EQEs of > 30% and LT95 of 140 h.^[^ ^27^, ^32^ ^]^ In such PHA molecules, the SOC is usually attributed to the mixing between ππ* and nπ* configurations in the excited states, as the carbonyl group possesses non‐bonding (n) orbitals,^[^ ^32^ ^]^ thus resulting in more spin‐allowed transitions according to El‐Sayed's rule and accelerating the RISC process. Since the nπ* configuration brought by lone‐pair electrons of oxygen atom typically has higher energy than the ππ* configuration, such a nπ*‐ππ* mixing is not easily feasible in most of the carbonyl‐embedded emitters. Therefore, the introduction of carbonyl units does not necessarily guarantee large SOC,^[^ ^23^, ^33^, ^34^ ^]^ and the number of carbonyl‐embedded PHA molecules with significant SOCs thus still remains limited. *Therefore, it is crucial to strengthen our understanding of the SOC mechanism and its relationship with the molecular structure in PHA emitters with a fused π‐conjugated framework*.

**Figure 1:**
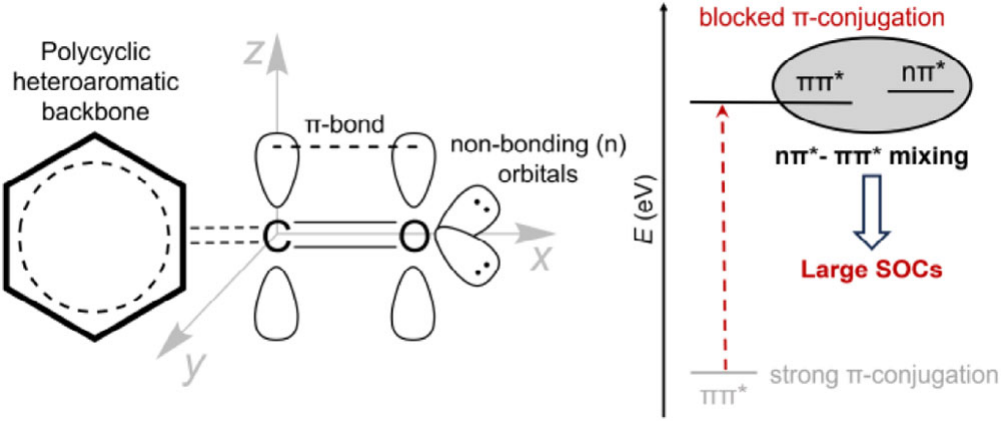
A schematic depiction of the potential approach to elevate energy of ππ^*^ configuration by blocking π‐conjugation in π‐fused polycyclic heteroaromatic emitters and thus inducing nπ^*^‐ππ^*^ mixing, which ultimately results in enhanced spin‐orbit couplings (SOCs).

As the size of π‐conjugated system increases, the optical band gap and thus ππ* excitation energy decreases.^[^ ^35^ ^]^ Even in carbonyl‐embedded molecules, the PHA backbone is essentially composed of π‐conjugated B,N, and C atoms. The ππ* excitation, derived from the orbital overlap between *p*_z_ orbitals of adjacent atoms in conjugated system, is delocalized across this whole backbone. This significant overlap usually leads to an enhancement of π molecular orbital energy and a decrease in π* molecular orbital energy, resulting in lower ππ* excitation energy. In contrast, the non‐bonding lone‐pair orbitals (n) localized on O atoms have a different spatial orientation, leading to lower overlap with the n and π* orbitals (Figure 1). Consequently, in π‐conjugated systems, a more localized nπ* transition occurs at a higher energy compared to the delocalized ππ* excitation, and the desired nπ*‐ππ* mixing thus remains limited despite the presence of carbonyl groups (Figure 1).

**Figure 2:**
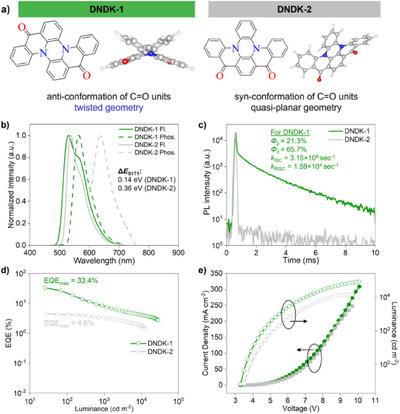
a) Chemical structures and equilibrium geometries of isomers DNDK‐1 and DNDK‐2 designed in this study. b) Fluorescence and phosphorescence spectra of 2.0 wt.% DNDK‐1/2‐doped mCP films at 77 K. c) Transient PL decay curve of 2 wt.% DNDK‐1/2‐doped mCP film and the associated kinetic parameters for DNDK‐1 (Φ_p_: the prompt fluorescence quantum efficiency; Φ_d_: the delayed fluorescence quantum efficiency; k_ISC_: rate of intersystem crossing). d) External quantum efficiency (EQE) versus luminance characteristics and e) Current density‐voltage‐luminance curves of the OLEDs based on DNDK‐1 and DNDK‐2.

Thus, in order to energetically elevate the ππ* configuration and potentially facilitate nπ*‐ππ* mixing, it is understandable to block π‐conjugation, which is associated with reducing the overlap between π‐orbitals, as shown in Figure 1. Based on this understanding, blocking π‐conjugation in π‐extended PHA backbones is thus necessary. In classic B/N‐embedded PHA emitters, the hybrid excitation of ππ* and πσ* induced by the distorted fused π‐conjugated structure was reported to enhance SOCs and RISC rates.^[^ ^2^ ^]^ Therefore, in the present work, to address the aforementioned problem, *we propose a potential approach*, i.e.*, blocking orbital π‐conjugation* via *either molecular‐structure distortion or meta‐arrangement of carbonyl‐π‐carbonyl and N‐π‐N, to boost SOC values in carbonyl‐embedded PHA emitters*. To explore the effect of blocking π‐conjugation through molecular‐structure distortion, we first synthesized two proof‐of‐concept isomeric carbonyl‐ and N‐embedded PHA compounds, named DNDK‐1 and DNDK‐2. DNDK‐1 exhibits a heavily twisted geometry, while DNDK‐2 attains a quasi‐planar structure (Figure 2a). Our experimental measurements have suggested a stark difference in their photophysical properties, with DNDK‐1 having relatively fast *k*_RISC_ (>10^4^ sec^−1^). Surprisingly, DNDK‐2 lacks any TADF property. Our high‐level quantum‐chemistry calculations have demonstrated that compared to DNDK‐2, DNDK‐1 not only exhibits reduced Δ*E*_S1T1_ and T_1_‐T_2_ energy gap (Δ*E*_T1T2_) but also has substantially larger SOC_S1T2_, eventually contributing to its higher *k*_RISC_. The twisted geometry in DNDK‐1 creates a hindrance in π‐conjugation across its backbone; the ππ* configuration with the elevated energy hybridizes with the nπ* configuration, thus significantly increasing the SOC values. Moreover, an extension of our observations from DNDK‐1/2 to two existing carbonyl‐ and N‐embedded isomeric molecules identified that in addition to molecular twisting, attenuation in π‐conjugation can also be achieved by utilizing the groups composed of meta‐arranged carbonyl‐Ar‐carbonyl and meta‐arranged N‐Ar‐N units. Our work has not only uncovered the role of hindered π‐conjugation in enhancing SOCs in carbonyl‐embedded PHA emitters but also provided a deep insight into the structure‐property relationship on SOC, which is thus helpful for further designing more PHA‐based TADF‐OLED emitters with high‐efficiency and low‐efficiency roll‐off driven by large SOCs.

## Results and Discussion

### Blocking Orbital π‐Conjugation via a Highly Twisted Molecular Structure

First, we designed two molecular isomers, DNDK‐1 and DNDK‐2, by arranging the C═O groups on different orientations in the π‐fused molecular skeleton. In DNDK‐1, the two C═O units are positioned on opposite sides (anti‐conformation), while in DNDK‐2, they remain on the same sides (syn‐conformation), as depicted in Figure 2a. As a result, DNDK‐1 has a heavily twisted structure, and DNDK‐2 adopts a quasi‐planar geometry.

To assess the effects of twisted and planar geometries on photophysical and optical properties, we synthesized the isomers DNDK‐1 and DNDK‐2 (Scheme S1), and their absorption and emission spectra were measured. Both DNDK‐1/2 display green emission in dilute toluene solution with emission maxima at 503/520 nm (Figure S3). A small FWHM of 39 nm in DNDK‐1 compared to 66 nm for DNDK‐2 indicates the presence of a lower degree of excited‐state geometrical reorganization in DNDK‐1 (Table S1). The prompt fluorescence and phosphorescence spectra of 2.0 wt.% DNDK‐1/2 doped mCP films at 77 K were also recorded, and S_1_ (T_1_) states of DNDK‐1 and DNDK‐2 were determined to be at 2.34 (2.20) and 2.31 (1.95) eV, respectively, leading to the corresponding Δ*E*_S1T1_ values of 0.14/0.36 eV for DNDK‐1/2 (Figure 2b). Moreover, our experiments on the transient PL decay properties of 2 wt.% DNDK‐1/2‐doped mCP films reveal a remarkable difference between the TADF properties of two isomers. The results in Figure 2c clearly depict the delayed‐fluorescence feature in DNDK‐1‐doped film with a fitted *k*_RISC_ of 1.6 × 10^4^ sec^−1^. From ambient air to nitrogen environment, the experimentally measured PLQY for DNDK‐1 was enhanced from 69% to 87%, while for DNDK‐2, no significant change was observed. Thus, the experimental results clearly demonstrate that altering the position of substituents and molecular‐structure planarity has astonishing effects on the optical properties of the two PHA emitters.

In view of the contrasting photophysical properties of DNDK‐1/2, we fabricate corresponding OLEDs with the following configurations: indium tin oxide (ITO)/4,4′‐(cyclohexane‐1,1‐diyl)bis(N,N‐di‐p‐tolylaniline) (TAPC, 30 nm)/tris(4‐(9H‐carbazol‐9‐yl)phenyl)amine (TCTA, 10 nm)/mCP:x wt.% DNDK‐1/‐2 (20 nm)/3,3′‐(5′‐(3‐(pyridin‐3‐yl)phenyl)‐[1,1′:3′,1″‐terphnyl]‐3,3″‐diyl)dipyridine (TmPyPB, 40 nm)/LiF (1 nm)/Al, and with the optimized doping concentration of 2.5 wt.% for each emitter. The devices have their emission peaks at 516 and 534 nm for DNDK‐1 and DNDK‐2, respectively, resulting in green OLEDs. As expected from the high k_RISC_ for DNDK‐1, its device achieves a maximum external quantum efficiency (EQE_max_) of 33.4%. On the other hand, DNDK‐2‐based device only achieves EQE_max_ of 4.6%.

**Figure 3:**
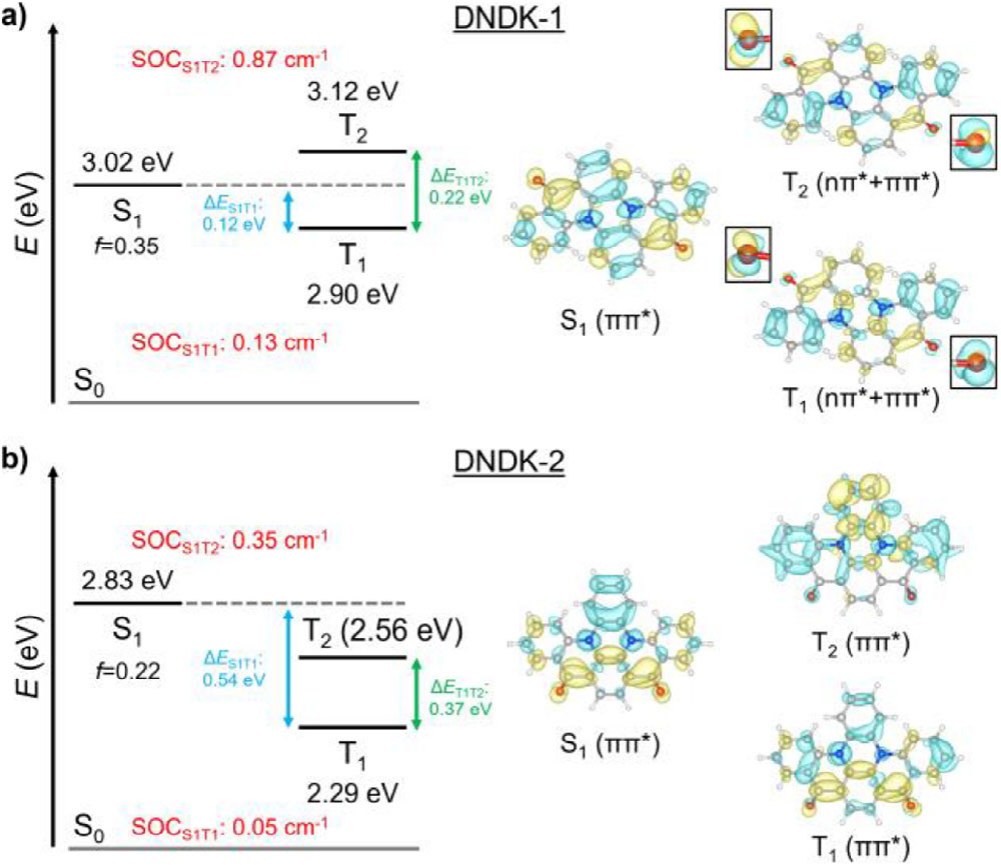
Excited‐state properties and corresponding difference density plots for a) DNDK‐1 and b) DNDK‐2 calculated at STEOM‐DLPNO‐CCSD level of theory; the excited state energy gaps (in eV), spin‐orbit couplings (SOCs, in cm^−1^), and oscillator strengths (*f*) of S_0_→S_1_ transition are labeled. A closer look at the different densities localized on C═O units is shown in the insets. The term in brackets refers to the excitonic nature of the excited state. The yellow and blue colors represent regions of increased and decreased electron density upon excitation, respectively.

To understand the origin of more significant TADF property and higher *k*_RISC_ in DNDK‐1 over DNDK‐2, we carried out high‐level quantum chemistry calculations. Due to the inability of popular time‐dependent density functional theories to accurately estimate the Δ*E*_S1T1_ in these PHA systems (Table S2), we examined the excited states of DNDK‐1/2 by employing STEOM‐DLPNO‐CCSD methodology based on coupled cluster theory which has proven to be very useful in understanding the electronic structure properties of excited states of PHA molecules.^[^ ^14^, ^16^, ^32^ ^]^ In Figure 3, our computational results reveal a remarkable difference in the excited‐state properties of the DNDK‐1 and ‐2 isomers. DNDK‐1 exhibits narrow energy gaps between its excited states, with Δ*E*_S1T1_ and Δ*E*_T1T2_ of 0.12 and 0.22 eV with significant SOC_S1T1_ and SOC_S1T2_ of 0.13 and 0.87 cm^−1^, respectively (Figure 3a). In contrast, Δ*E*_S1T1_ and Δ*E*_T1T2_ for DNDK‐2 are much larger at 0.54 and 0.37 eV, with only marginal SOC_S1T1_ and SOC_S1T2_ of 0.05 and 0.35 cm^−1^, respectively (Figure 3b). Our high‐level calculation results on the energies of their S_1_ and T_1_ states as well as the Δ*E*_S1T1_ values, are qualitatively consistent with the values obtained via the experimentally measured spectra. To assess the effects of these distinct energy gaps and SOC values on the RISC process, we further calculated the Boltzmann‐averaged *k*_RISC_ value of 1.4 × 10^5^ sec^−1^ for DNDK‐1, under the framework of Marcus equation based on time‐dependent perturbation theory (for computational details, see the “Quantum chemical calculations” section in the Supporting Information). The major contribution to *k*_RISC_ comes from the T_2_→S_1_ transition with the calculated *k*_T2→S1_ of 6.3 × 10^8^ sec^−1^, a much larger value as compared to the *k*_T1→S1_ of 1.1 × 10^4^ sec^−1^. In contrast, the calculated *k*_RISC_ value for DNDK‐2 is negligibly small, indicating no TADF property.

**Figure 4:**
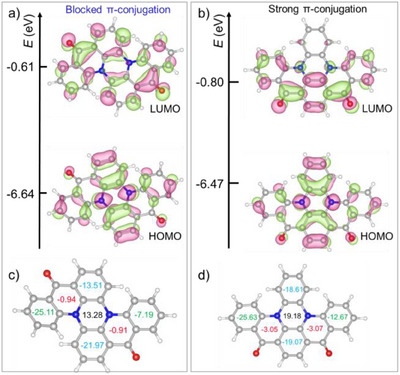
Frontier molecular orbitals for a) DNDK‐1 and b) DNDK‐2. NICS(1)_zz_ values for the equilibrium geometries of c) DNDK‐1 and d) DNDK‐2. Values labeled in black, blue, red, and green represent the central, top/bottom, carbonyl‐substituted, and left/right rings, respectively.

To understand the above differences in the electronic structures of the excited states in DNDK‐1 and DNDK‐2, their frontier molecular orbitals are first examined, since electronic structure of excited state is determined by frontier molecular orbitals involved in electronic excitation. Interestingly, an inspection of the frontier molecular orbitals of DNDK‐1/2 unveils a fundamental difference due to the molecular‐structure distortion caused by the orientation of C═O groups. The significant distortion of the molecular structure hinders the π‐conjugation in DNDK‐1 backbone, while the π‐conjugation in the quasi‐planar DNDK‐2 remains stronger, which is reflected by the larger energy gap of 6.03 eV between HOMO and LUMO orbitals in DNDK‐1 than that of 5.67 eV in DNDK‐2 (Figure 4a,b). Then, we examined the compositions of the excited states for DNDK‐1 and DNDK‐2 (see Figure S5). For DNDK‐1, all the S_1_ and T_1_ states are composed of the multiple electronic transitions, including HOMO→LUMO and higher‐energy HOMO→LUMO+1. Both its S_1_ and T_1_ states are similarly mainly composed of the HOMO→LUMO transition, with the small but non‐negligible contributions from the HOMO→LUMO+1 transitions. Therefore, the S_1_ and T_1_ states are close in energy, thus resulting in small ΔE_S1T1_ in DNDK‐1. For DNDK‐2, its S_1_ state has a main contribution from HOMO→LUMO accompanied by a small contribution of the higher‐energy HOMO‐1→LUMO+1. In contrast, the T_1_ state of DNDK‐2 is overwhelmingly composed of the low‐energy HOMO→LUMO. As previously discussed, the HOMO‐LUMO gap in DNDK‐2 is much smaller due to the strong π‐conjugation of its planar structure. Such lower‐energy HOMO‐LUMO gap eventually results in its lower‐energy T_1_ state, while the higher‐energy HOMO‐1→LUMO+1 composition of its S_1_ state has a compensation for its S_1_‐state energy, which contributes to a much larger ΔE_S1T1_ in DNDK‐2. Overall, the T₁ state of DNDK‐2 loses its multiconfigurational character, becoming predominantly confined to the HOMO→LUMO transition. This is the reason why in DNDK‐2 with a quasi‐planar structure, the T_1_‐state energy is significantly reduced with respect to that of DNDK‐1 with a twisted structure. The much smaller Δ*E*_T1T2_ in DNDK‐1 stems from its smaller LUMO  – LUMO+1 energetic splitting of only 0.22 eV, compared to 0.64 eV in DNDK‐2. In DNDK‐1, T_1_ and T_2_ have significant contributions from HOMO→LUMO (≈76%) and HOMO→LUMO+1 (≈61%) configurations, respectively; a smaller LUMO ̶ LUMO+1 splitting thus results in energetically close‐lying T_1_ and T_2_. On the other hand, T_1_ and T_2_ of DNDK‐2 are primarily composed of HOMO→LUMO (≈91%) and HOMO‐1→LUMO (≈84%), respectively; a wide HOMO  – HOMO‐1 splitting thus lead to large Δ*E*_T1T2_ (Figure S5). In order to visualize excited‐state characteristics, the difference density plots based on the STEOM‐DLPNO‐CCSD calculations are shown in Figure 3, suggesting that the triplet states, especially T_2_, of DNDK‐1 have a strong nπ*‐ππ* mixing, thereby establishing the involvement of different excitation configurations in the S_1_ and T_2_ states (Figure 3a). In contrast, both S_1_ and T_1_/T_2_ of DNDK‐2 are primarily ππ* states (Figure 3b). Therefore, the spin‐orbit interactions in DNDK‐1 are more prominent, especially for SOC_S1T2_, in comparison to its counterpart.

Furthermore, we calculate the nucleus‐independent chemical shifts (NICS), one of the most widely used tools to probe aromaticity and extent of π‐conjugation in aromatic molecules.^[^ ^36^ ^]^ Average NICS(1)_zz_ values, calculated 1 Å above and below the molecular plane, reflect the influence of π‐electrons derived from *p*_z_ orbitals, indicating the aromatic (negative values) or anti‐aromatic (positive values) nature of a conjugated ring. In Figure 4c,d, positive NICS(1)_zz_ values quantitatively confirm the anti‐aromatic nature of central rings in both DNDK‐1 and ‐2, with a smaller NICS(1)_zz_ of 13.28 in DNDK‐1 indicating hindrance in π‐conjugation compared to 19.18 in DNDK‐2. As the orientation of C═O units is primarily responsible for molecular twisting in DNDK‐1, its carbonyl‐substituted rings exhibit less negative NICS(1)_zz_ of −0.94/−0.91 compared to −3.05/−3.07 in quasi‐planar DNDK‐2. Moreover, the top/right rings in DNDK‐1 display less negative NICS(1)_zz_ of −13.51/−7.19 compared to −18.61/−12.67 in DNDK‐2, implying the blocked π‐conjugation across the backbone in DNDK‐1.

These results confirm that blocked π‐conjugation caused by the molecular distortion in DNDK‐1 eventually leads to the energetic elevation of the delocalized ππ* excitation.^[^ ^34^, ^37^ ^]^ Now, this ππ* excitation can mix with the high‐lying nπ* configuration (as schematically shown in Figure 1) and ultimately induce large SOCs in the DNDK‐1, which is observed in the difference density analysis of DNDK‐1. The triplet excited states (especially T_2_) in DNDK‐1 show the nπ* characters mixed with ππ* characters owing to its hindered π‐conjugation and thus have remarkable SOC_S1T2_, as discussed above in Figure 3. DNDK‐2, which lacks such a hindrance of π‐conjugation, does not have any prominent nπ*‐ππ* mixing and results in marginal SOC. These observations validate our hypotheses that if an appropriate energetic elevation of ππ* configuration can be achieved by blocking π‐conjugation, it is possible for the nπ* configurations to mix with the usual ππ* configurations in excited states, which ultimately results in significant SOCs, thus enhancing the RISC process. So, the fine‐tuning of π‐conjugation in the PHA backbone can be an efficient approach for designing more efficient PHA‐based TADF molecules.

### Blocking Orbital π‐Conjugation via Meta Substitutions

**Figure 5:**
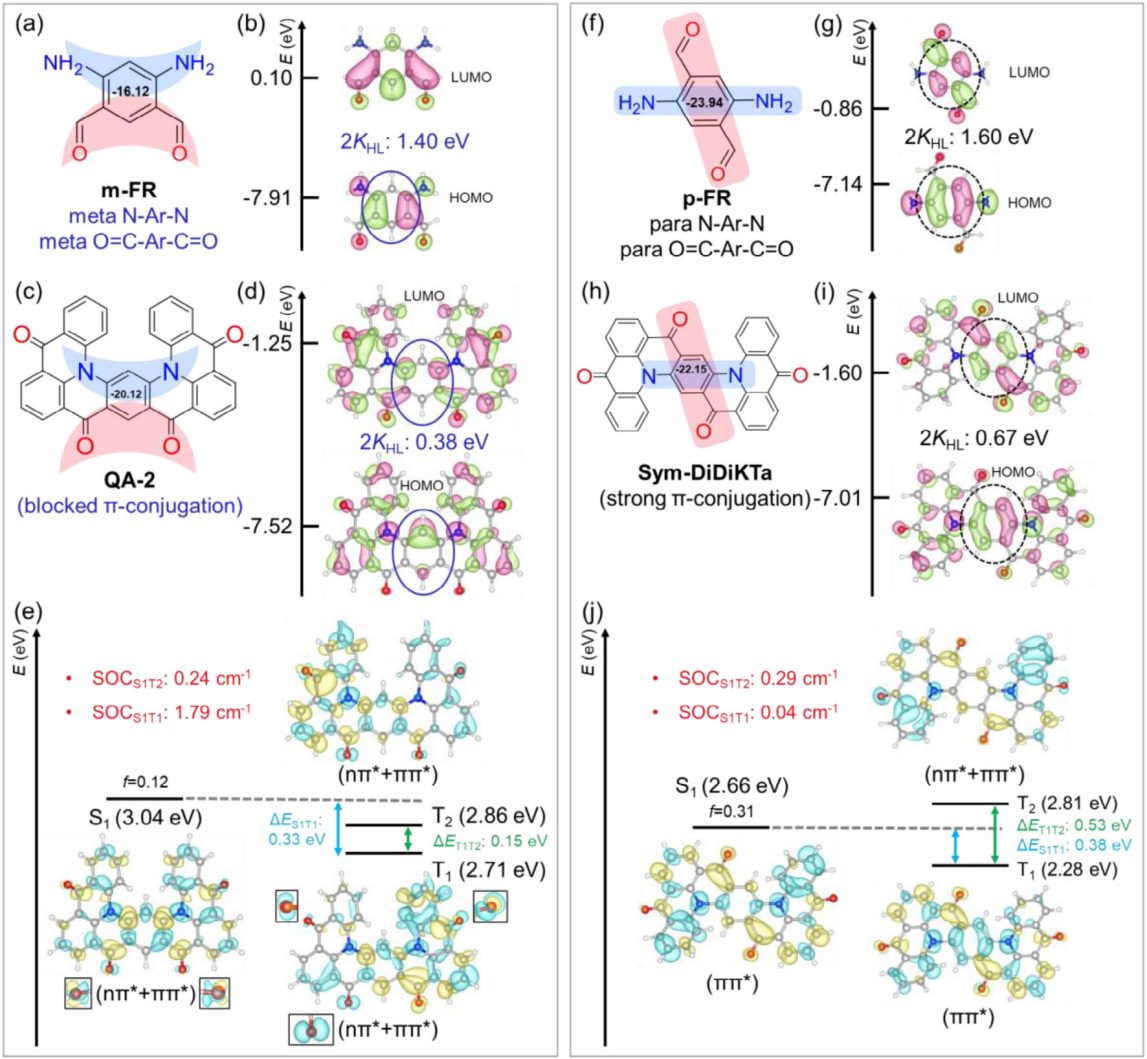
a) Chemical structure with NICS(1)_zz_ and b) frontier molecular orbitals of m‐FR. c) Chemical structure with NICS(1)_zz_ labeled on central ring, d) frontier molecular orbitals, and e) excited state properties with difference density analysis for QA‐2. f) Chemical structure labelled with NICS(1)_zz_ and g) frontier molecular orbitals of p‐FR. h) Chemical structure with NICS(1)_zz_ labeled on central ring, i) frontier molecular orbitals, and j) excited state properties with difference density analysis for Sym‐DiDiKTa. The region highlighted by blue solid eclipses represents the hindrance of π‐conjugation due to the non‐bonding nature of wavefunction; the black dashed eclipses highlight the bonding/anti‐bonding nature of wavefunction. *K*_HL_ is HOMO‐LUMO exchange energy, and Δ*E*/SOC corresponds to the energy gaps/spin‐orbit couplings among excited states. A closer look at the wave function localized on C═O units is shown in the insets. The term in brackets refers to the excitonic nature of a particular excited state.

In addition to the highly twisted geometrical structure, we recall from organic‐chemistry textbooks that the hindrance of π‐conjugation can also be achieved by utilizing the meta‐substituted groups. In a meta‐substituted molecule, there is a lack of direct resonance pathways between substituents, and orbital overlap is disrupted. However, the para‐substitution allows continuous π‐electron delocalization over the whole aromatic ring.^[^ ^38^ ^]^ Herein, we consider two model fragment structures, m‐FR and p‐FR, designed by using the meta and para‐arrangements of N‐Ar‐N and carbonyl‐Ar‐carbonyl units, respectively. The less negative NICS(1)_zz_ of −16.12, compared to −23.94 in p‐FR, confirms the hindrance in π‐conjugation due to meta‐arrangement (Figure 5a,f). Additionally, HOMO in m‐FR exhibit non‐bonding characteristics, respectively (highlighted by solid blue ellipses), indicating the hindrance of π‐conjugation (Figure 5b). In contrast, the central ring in p‐FR displays bonding/anti‐bonding features in its HOMO and LUMO (highlighted by dashed black ellipses), resulting in a smoother π‐conjugation (Figure 5g). As a result of the hindered π‐conjugation, the HOMO‐LUMO gap in m‐FR is significantly larger at 8.01 eV compared to 6.28 eV in p‐FR. Consequently, not only 2*K*_HL_ in m‐FR smaller, but its ππ* excitation configuration is also energetically elevated.

To further verify the observations from the discussion on m‐FR versus p‐FR, we examine QA‐2 (Figure 5c) and Sym‐DiDiKTa (Figure 5h) isomers as examples, which are quinacridone derivatives developed by Yasuda et al.^[^ ^22^ ^]^ and Zysman–Colman et al.,^[^ ^23^ ^]^ respectively. Similar to m‐FR, QA‐2 has a central moiety composed of meta‐arranged carbonyl‐Ar‐carbonyl and meta‐arranged N‐Ar‐N units. In contrast, the substituents in Sym‐DiDiKTa follow a para‐arrangement, akin to p‐FR.

In QA‐2, the meta‐substituted central moiety gives rise to the apparent non‐bonding feature of its HOMO and LUMO orbitals (see the regions highlighted by blue solid eclipses), suggesting the hindered π‐conjugation in the above molecular orbitals (Figure 5d). In contrast, in Sym‐DiDiKTa, the para‐substituted central moiety leads to the bonding/anti‐bonding feature of its HOMO/LUMO (see the regions highlighted by black dashed eclipses), implying a smooth π‐conjugation (Figure 5i) in the central ring. As a result of hindered π‐conjugation, the central ring in QA‐2 exhibits less negative NICS(1)_zz_ of −20.12 compared to that of −22.15 in Sym‐DiDiKTa (Figure 5c,h).

Unlike the DNDK‐1/2 case, the differences in the orbital π‐conjugation of QA‐2 and Sym‐DiDiKTa arise due to the arrangement of substituents rather than from their geometric orientations, as the planarity in these molecules is deformed to a similar extent (Figure S6). Driven by hindered π‐conjugation, the energy gap between the HOMO and LUMO, in QA‐2, is much larger (6.27 eV) than that in Sym‐DiDiKTa (5.41 eV), which is also reflected by its higher S_1_ and T_1_‐state energies discussed in Figure 5. Moreover, the non‐bonding fashion of the HOMO and LUMO orbitals in QA‐2 hinders their spatial overlap (*O*_HL_ value of 0.61 as compared to 0.65 for Sym‐DiDiKTa) and thus leads to a smaller 2*K*_HL_ value of 0.38 eV, compared to 0.67 eV in Sym‐DiDiKTa. Consequently, the calculated Δ*E*_S1T1_ (0.33 eV) in QA‐2 is smaller than that of 0.38 eV in Sym‐DiDiKTa. Moreover, Δ*E*_T1T2_ of 0.15 eV in QA‐2 is nearly one‐third smaller than that in Sym‐DiDiKTa (Figures 5e,j). The proximal T_1_ and T_2_ states in QA‐2 are direct implications of close‐lying molecular orbitals, as the energetic separation between HOMO/HOMO‐1 and LUMO/LUMO+1 pairs is only of 0.06 and 0.16 eV, as compared to that of 0.71 and 0.64 eV in Sym‐DiDiKTa, respectively. Therefore, both T_1_ and T_2_ in QA‐2 have significant contributions from all HOMO‐1, HOMO, LUMO and LUMO+1 orbitals, while they are primarily confined to only HOMO→LUMO and HOMO‐1→LUMO+1 configurations in Sym‐DiDiKTa (Figure S7).

The hindered π‐conjugation in QA‐2 causes the elevation of ππ* excitation energy as compared to Sym‐DiDiKTa (Figure 5d,i). This enables the mixing between nπ^*^ and ππ^*^ configurations in QA‐2 as evident from the S_1_/T_1_ difference density plots (Figure 5e), which yields a significant SOC_S1T1_ of 1.79 cm^−1^. In contrast, the para‐arrangement in Sym‐DiDiKTa results in a very small SOC_S1T1_, as no nπ*‐ππ* mixing occurs in its excited states (Figure 5j). Driven by its larger SOC_S1T1_ and smaller Δ*E*_S1T1_/Δ*E*_T1T2_, QA‐2 becomes a favorable candidate over Sym‐DiDiKTa to achieve faster *k*_RISC_ and delayed fluorescence decay rate (*k*_d_). Our results are further validated by previously reported experimental explorations on QA‐2 and Sym‐DiDiKTa, with the doped film of QA‐2 exhibiting a much shorter delayed fluorescence lifetime (*τ*_d_) of 48 µs^[^ ^22^ ^]^ compared to that of Sym‐DiDiKTa (*τ*_d _= 1700 µs).^[^ ^23^ ^]^ *These results demonstrate that the alteration in π‐conjugation can be an effective tool in enhancing the SOCs. In addition to employing twisted molecular structures, the analysis of QA‐2 *versus *Sym‐DiDiKTa validates the alternative way of modulating the π‐conjugation by introducing the meta‐substituted moieties and capitalizing on their electronic effects*.

Despite the presence of C═O groups in DNDK‐2 and Sym‐DiDiKTa, there is only little nπ*‐ππ* mixing in their singlet and triplet states, and the SOC_S1T1_ values remain vanishingly small. Therefore, the presence of C═O groups does not guarantee a mixing between ππ* and nπ* configurations unless there is an appropriate energy matching between these two configurations. Since the nπ* configuration with a localized lone‐pair orbital usually has a high energy, the energy of ππ* configuration should be appropriately elevated for a favorable nπ*‐ππ* mixing. As discussed above for DNDK‐1 and QA‐2, this can be achieved by creating a hindrance in orbital π‐conjugation of the PHA backbones.

**Figure 6:**
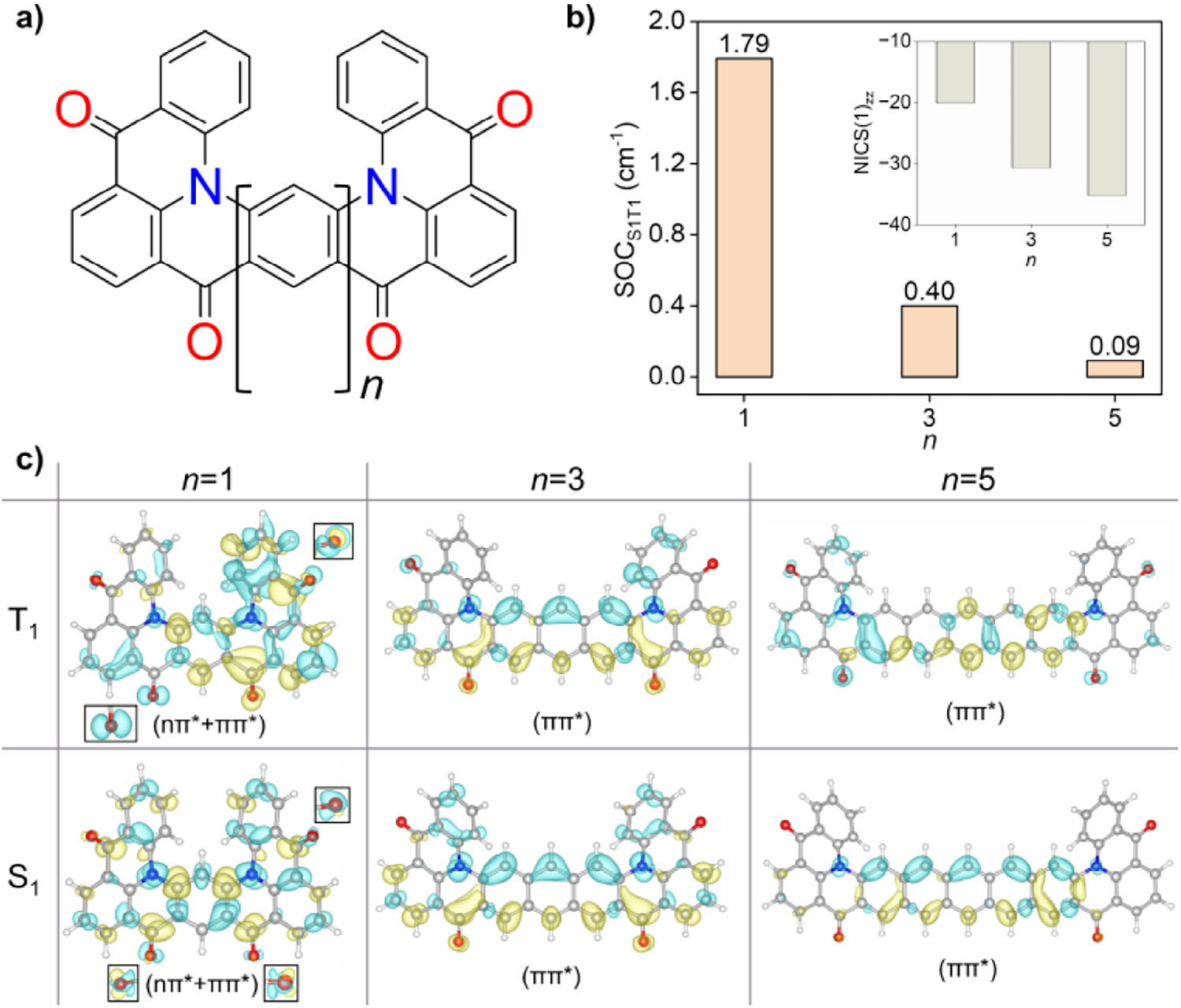
a) The extension of π‐conjugation in QA‐2*_n_* by a factor *n* that relates to the number of central rings. b) SOC_S1T1_ plotted as a function of *n* with NICS(1)_zz_ values on central benzene rings versus *n*. c) Difference densities of S_1_ and T_1_ states of QA‐2*_n_* molecules as *n *= 1,3 and 5. A closer look at the different densities localized on C═O units is shown in the insets. The yellow and blue colors represent regions of increased and decreased electron density upon excitation, respectively.

To demonstrate the critical correlation among the extent of orbital π‐conjugation, nπ*‐ππ* mixing, and SOC, we take QA‐2 as an example and systematically extend the extent of conjugation in QA‐2*_n_* by a factor *n* that relates to the number of central benzene rings, as shown in Figure 6a. As the extent of π‐conjugation becomes increasingly large, depicted by gradually more negative NICS(1) values of −20.12, −30.69, −35.26 on central rings with increasing *n*, the SOC_S1T1_ gradually decreases and becomes negligible for QA‐2*_n_*_= 5_ (Figure 6b). At highly conjugated structures, e.g., QA‐2*_n_*_= 5_, the ππ* characters dominate their S_1_ and T_1_ states. However, as the orbital π‐conjugation is hindered by reducing the number of conjugated rings in QA‐2*_n_*_= 1_, the S_1_ and T_1_ states possess considerable nπ* characters together with the ππ* characters, as the energetic elevation of ππ* configuration now facilitates the mixing (Figure 6c). In the absence of such elevation in ππ* energy, the nπ*‐ππ* mixing does not emerge, and SOCs are attenuated. It is straightforward to comprehend that the hindrance of π‐conjugation is accompanied by an increase in the HOMO‐LUMO energy gap, which elevates the ππ* energy (Figure S8). In addition, if the π‐conjugation is significantly hindered and the nπ* character is prominently mixed in the S_1_ state, the spin‐flip between nπ* and ππ* configurations is possible. However, this typically results in a negative outcome on the fluorescent oscillator strengths (*f*) of MR molecules. For example, the nπ* mixing in S_1_ of QA‐2 significantly reduces *f* to a smaller value of 0.12, as compared to its counterpart Sym‐DiDiKTa, which has no nπ* mixing, thus *f *= 0.31 (Figure 5). Therefore, it is crucial to finely tune the orbital π‐conjugation in the PHA backbone to maintain a balance between SOCs and oscillator strength, thus ensuring that S_1_ has the desired photophysical properties.

## Conclusion

Our experimental and theoretical explorations provide a deep understanding of the origin of spin‐orbit coupling and its structure‐property relationship in several carbonyl‐embedded polycyclic heteroaromatic molecules. We proposed and validated blocking π‐conjugation in these molecules as an effective approach to induce nπ*‐ππ* mixing, enhance SOCs, and improve RISC rates in MR materials. The hindrance of the orbital π‐conjugation due to its twisted polycyclic heteroatomic backbone in DNDK‐1 increases its HOMO‐LUMO energy gap and eventually elevates the ππ* configuration energy, which facilitates the hybridization of ππ* with nπ* configuration in its excited states. According to the El‐Sayed rule, different excitation characters of the singlet and triplet excited states in DNDK‐1 yield significant spin‐orbit coupling that is necessary for an efficient RISC and, thus, the TADF process. In another quasi‐planar isomer, DNDK‐2, a much stronger π‐conjugation stabilizes its ππ* configuration, and spin‐orbit couplings thus remain marginal. The extension of these observations to the other existing molecular isomers, QA‐2 and Sym‐DiDiKTa, paves another interesting pathway to hindering the π‐conjugation by introducing the electron‐rich and electron‐deficient substituents in a meta‐arranged carbonyl‐Ar‐carbonyl and *meta*‐arranged N‐Ar‐N fashion, thus enhancing SOCs. The key factor here is to finely tune the π‐conjugation *either by molecular‐structure distortion or by employing meta‐arrangement of chemical substituents* to elevate the ππ* configuration energy and induce the ππ*‐nπ* mixing in excited states, which eventually leads to enhanced spin‐orbit couplings. Furthermore, our results show that in the absence of such hindered π‐conjugation, the spin‐orbit couplings are suppressed. Our work will thus facilitate the design of more efficient polycyclic heteroatomic emitters with faster RISC rates driven by the large SOCs.

## Supporting Information

Supporting Information is available from the Wiley Online Library or the corresponding authors. The authors have cited additional references within the Supporting Information.^[^ ^39^, ^40^, ^41^, ^42^, ^43^ ^]^

## Conflict of Interests

The authors declare no conflict of interest.

## Supporting information



Supporting Information

## Data Availability

The data that support the findings of this study are available from the corresponding author upon reasonable request.
