# Supplementary material for: Blocking Orbital π‐Conjugation to Boost Spin‐Orbit Coupling in Carbonyl‐Embedded Polycyclic Heteroaromatic Emitters
Source: Angew Chem Int Ed Engl. 2025 Mar 22;64(19):e202503371. doi: 10.1002/anie.202503371 (PMC12051818; doi:10.1002/anie.202503371)
Supplement: Supplementary file 1 — Supporting Information [file ANIE-64-e202503371-s001.docx]

Supporting Information

**Blocking Orbital π-Conjugation to Boost Spin-Orbit Coupling in Carbonyl-Embedded Polycyclic Heteroatomic Emitters**

Rajat Walia^1,#^, Xiaochun Fan^1,2,#^, Le Mei^3^, Weixiong Guo^3^, Kai Wang^1,4^, Chihaya Adachi*^,2^, Xian-Kai Chen*^,1,2,5^, and Xiao-Hong Zhang^1,5^

^1^ Institute of Functional Nano and Soft Materials (FUNSOM), Joint International Research Laboratory of Carbon-Based Functional Materials and Devices, Soochow University, Suzhou, Jiangsu, People’s Republic of China.

^2^ Center for Organic Photonics and Electronics Research (OPERA), Kyushu University,

15 Fukuoka, 819-0395, Japan

^3^ Department of Chemistry, City University of Hong Kong, Kowloon, Hong Kong SAR, People’s Republic of China.

^4^ Jiangsu Key Laboratory for Carbon-Based Functional Materials & Devices, Soochow University, 215123 Suzhou, Jiangsu, People’s Republic of China.

^5^ Jiangsu Key Laboratory of Advanced Negative Carbon Technologies, Soochow University, Suzhou, Jiangsu, People’s Republic of China.

# These authors contributed equally to this work.

**Email:** adachi@cstf.kyushu-u.ac.jp (C.A.); [xkchen@suda.edu.cn](mailto:xkchen@suda.edu.cn) (X.-K.C.)

**Quantum chemical calculations:** To obtain the ground-state equilibrium geometries for all molecules, the range-separated hybrid density functional ωB97XD with the nonempirically tuned ω value was employed with Gaussian 16 software.^[1]^ Based on the optimized geometries, the STEOM-DLPNO-CCSD calculations were carried out in ORCA 4.2.1 quantum chemistry package.^[2]^ The HOMO-LUMO exchange energy was calculated using PySCF package.^[3]^ The NICS(1)_zz_ values were calculated utilizing the procedure described in Multiwfn.^[4]^ The compositions of the excited states were evaluated by correlating four electrons in four active orbitals (HOMO-1, HOMO, LUMO and LUMO+1) within the complete active space self-consistent field (CASSCF) method. The def2-TZVP^[5]^ basis set was employed for all calculations.

We employed the Marcus theory based on Fermi’s golden rule and calculated the rate constants *k*_T1→S1_ and *k*_T2→S1_ related to T_1_→S_1_ and T_2_→S_1_ processes, respectively,

$$k_{T_{1/2}\to S_{1}}=\frac{2\pi}{\hbar}{SOC(T_{1/2}-S_{1})}^{2}\frac{1}{\sqrt{4\pi\lambda_{T_{1/2}-S_{1}}k_{B}T}}\exp\left( -\frac{{(E_{S_{1}}-E_{T_{1/2}}+\lambda_{T_{1/2}-S_{1}})}^{2}}{4k_{B}T\lambda_{T_{1/2}-S_{1}}} \right)$$

and, then, using the Boltzmann-averaging to evaluate the final RISC rate,

$${k_{\mathrm{RISC}}=\frac{e^{-\frac{E_{T_{1}}}{k_{B}T}}}{e^{-\frac{E_{T_{1}}}{k_{B}T}}+e^{-\frac{E_{T_{2}}}{k_{B}T}}}k}_{T_{1}\to S_{1}}{+\frac{e^{-\frac{E_{T_{2}}}{k_{B}T}}}{e^{-\frac{E_{T_{1}}}{k_{B}T}}+e^{-\frac{E_{T_{2}}}{k_{B}T}}}k}_{T_{2}\to S_{1}}$$

## here, k_B_ represents the Boltzmann constant; T is the temperature; and $\boldsymbol{\lambda}_{\boldsymbol{T}_{\boldsymbol{1/2}}\boldsymbol{-}\boldsymbol{S}_{\boldsymbol{1}}}$ denotes the reorganization energy related to the T_1_/T_2_→S_1_ transition.

## Photophysical measurements: Ultraviolet–visible absorption spectra were recorded on a Hitachi U-3900 spectrophotometer. Fluorescence and phosphorescence spectra were recorded on a Hitachi F-4600 fluorescence spectrophotometer. TR-PL decay curves were measured with FLS-980 Series of Fluorescence Spectrometers. Absolute PLQYs of the doped films were recorded on a Hamamatsu Quantaurus-QY quantum yield spectrometer (C13534-11) under air/nitrogen conditions.

**Molecular synthesis and characterization:** All reagents were purchased from commercial sources and used without further purification. ^1^H NMR and ^13^C NMR spectra were recorded on a Bruker 400/600/101 MHz spectrometer in a suitable deuterium reagent at room temperature. MALDI-TOF mass data were recorded on a Bruker ultrafleXtreme instrument.

**Scheme S1 |** Synthetic routes of DNDK-1 and DNDK-2.

**Synthesis of dimethyl 2,2'-(phenazine-5,10-diyl)dibenzoate (2).** Compound 1 (1.8 g, 10 mmol), methyl 2-bromobenzoate (4.7 g, 22 mmol), Pd(OAc)_2_ (135 mg, 0.6 mmol), *x*Phos (571 mg, 1.2 mmol), and Cs_2_CO_3_ (9.8 g, 30 mmol ) were dissolved in *o*-xylene (200 mL). The mixture was degassed by a vacuum-nitrogen cycle and heated to reflux for 24 hours. After cooling it to room temperature, the reaction mixture was filtered with a pad of silica gel and concentrated in *vacuo*. The crude product was further purified by column (PE: DCM = 1:4) and dried under vacuum to obtain dark-red solid compound 2 (2.5 g, 56%). ^1^H NMR (600 MHz, CDCl_3_, ppm) δ 8.15 (d, J = 7.7 Hz, 2H), 7.76 (t, J = 7.7 Hz, 2H), 7.54 (t, J = 7.7 Hz, 2H), 7.45 (d, J = 7.8 Hz, 2H), 6.19 (s, 4H), 5.44 (s, 4H), 3.89 (s, 6H). MS (MALDI-TOF). Calcd for C_28_H_22_N_2_O_4_: 450.49; Found: 450.48.

**Synthesis of 2,2'-(phenazine-5,10-diyl)dibenzoic acid (3).** Compound 2 (2.3 g, 5 mmol) and NaOH (2.0 g, 50 mmol) were dissolved in 1,4-dioxane (50 mL) and water (50 mL). The mixture was heated to reflux for 12 hours. After cooling it down to room temperature, the reaction mixture was poured into water (200 mL) and acidified with dilute hydrochloric acid to a pH of 2~3. The suspension was filtered and washed with plenty of water. The crude product was further dried under vacuum to obtain dark-red solid compound 3 (1.9 g, 90%) and used without further purification.

**Synthesis of 9*H*,18*H*-diquinolino[3,2,1-*de*:3',2',1'-*kl*]phenazine-9,18-dione (DNDK-1) and diquinolino[3,2,1-*de*:1',2',3'-*mn*]phenazine-5,8-dione (DNDK).** A mixture of compound 3 (1.7 g, 4 mmol) and 10 mL polyphosphoric acid (PPA) was slowly stirred and heated to 140 °C under a nitrogen atmosphere for 48 h. After cooling it to room temperature, the reaction mixture was diluted with 100 mL water and carefully neutralized with aqueous sodium hydroxide in an ice-water bath. Then, the suspension was filtered and further purified by column (DCM) and dried under vacuum to obtain orange solid DNDK-1 (773 mg, 50%) and DNDK-2 (340 mg, 22%). DNDK-1: ^1^H NMR (400 MHz, CD_2_Cl_2_, ppm) δ 8.50 (dd, J = 8.1, 1.7 Hz, 2H), 8.12 (d, J = 7.9 Hz, 2H), 8.06 (d, J = 8.6 Hz, 2H), 7.81 (dd, J = 7.9, 1.3 Hz, 2H), 7.74 (ddd, J = 8.7, 7.1, 1.7 Hz, 2H), 7.48 (t, J = 7.5 Hz, 2H), 7.37 (t, J = 8.0 Hz, 2H). ^13^C NMR (101 MHz, CD_2_Cl_2_, ppm) δ 177.06, 137.57, 133.01, 131.55, 127.56, 125.36, 124.52, 124.11, 123.49, 122.19, 120.99, 118.22. MS (MALDI-TOF). Calcd for C_26_H_14_N_2_O_2_: 386.41; Found: 386.41. DNDK-2: ^1^H NMR (400 MHz, CD_2_Cl_2_, ppm) δ 8.52 (dd, J = 8.1, 1.2 Hz, 2H), 8.27 (dt, J = 8.6, 0.7 Hz, 2H), 8.13 (s, 2H), 7.85 - 7.79 (m, 4H), 7.54 (ddd, J = 8.1, 7.1, 1.0 Hz, 2H), 7.31 (dd, J = 6.3, 3.4 Hz, 2H). ^13^C NMR (101 MHz, CD_2_Cl_2_, ppm) δ 176.96, 138.34, 135.38, 132.69, 132.65, 127.20, 126.00, 125.83, 124.35, 122.99, 120.27, 119.60, 119.36. MS (MALDI-TOF). MS (MALDI-TOF). Calcd for C_26_H_14_N_2_O_2_: 386.41; Found: 386.39. Crystals of DNDK-1 and DNDK-2 were both obtained via liquid phase diffusion in chloroform and ethyl acetate. [CCDC 2166866 (DNDK-1) and 2166867 (DNDK-2) contain the supplementary crystallographic data for this paper. These data can be obtained free of charge from The Cambridge Crystallographic Data Centre via www.ccdc.cam.ac.uk/data_request/cif.].


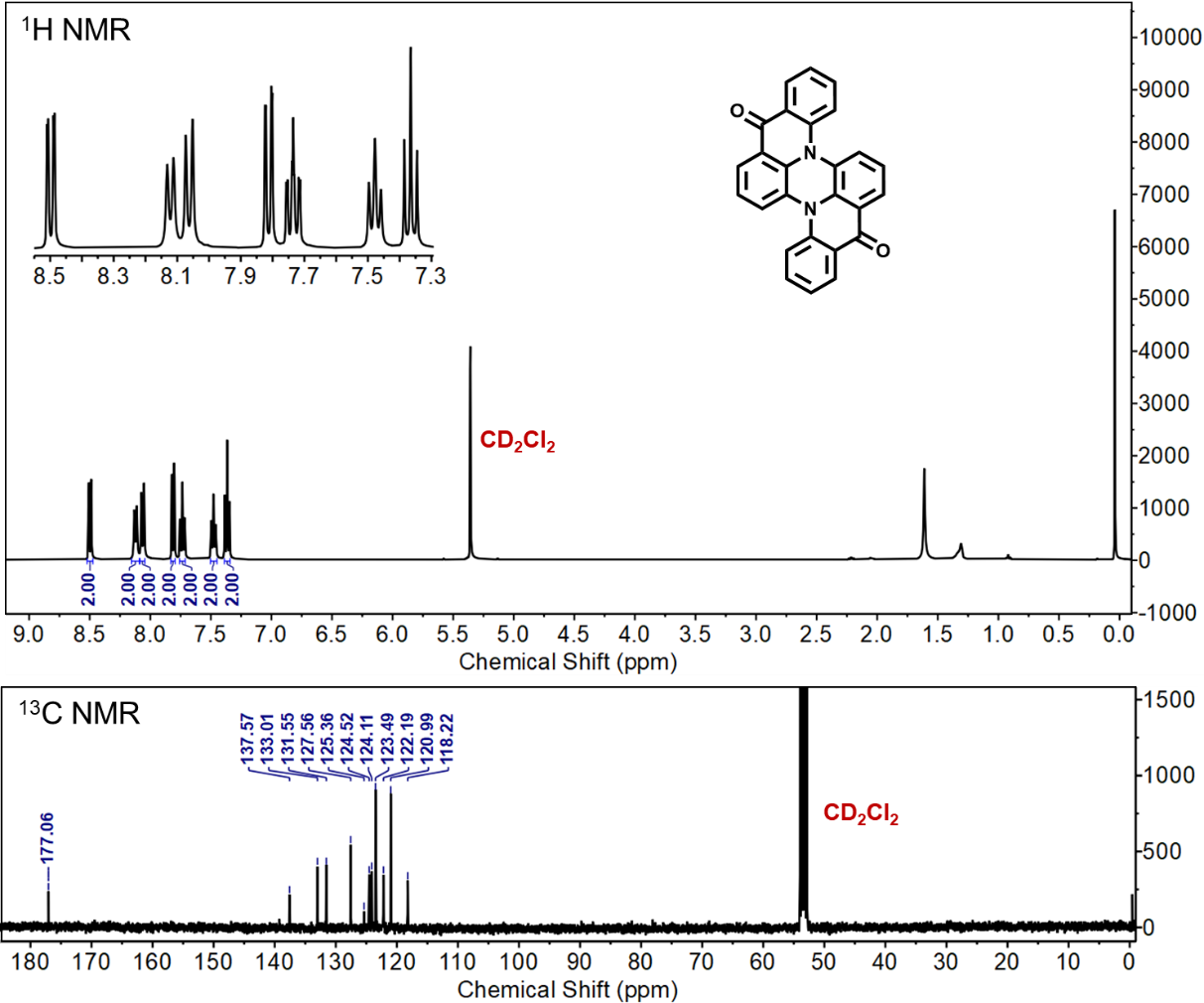


**Figure S1**. ^1^H and ^13^C NMR spectra of DNDK-1 in CD_2_Cl_2_ at room temperature.


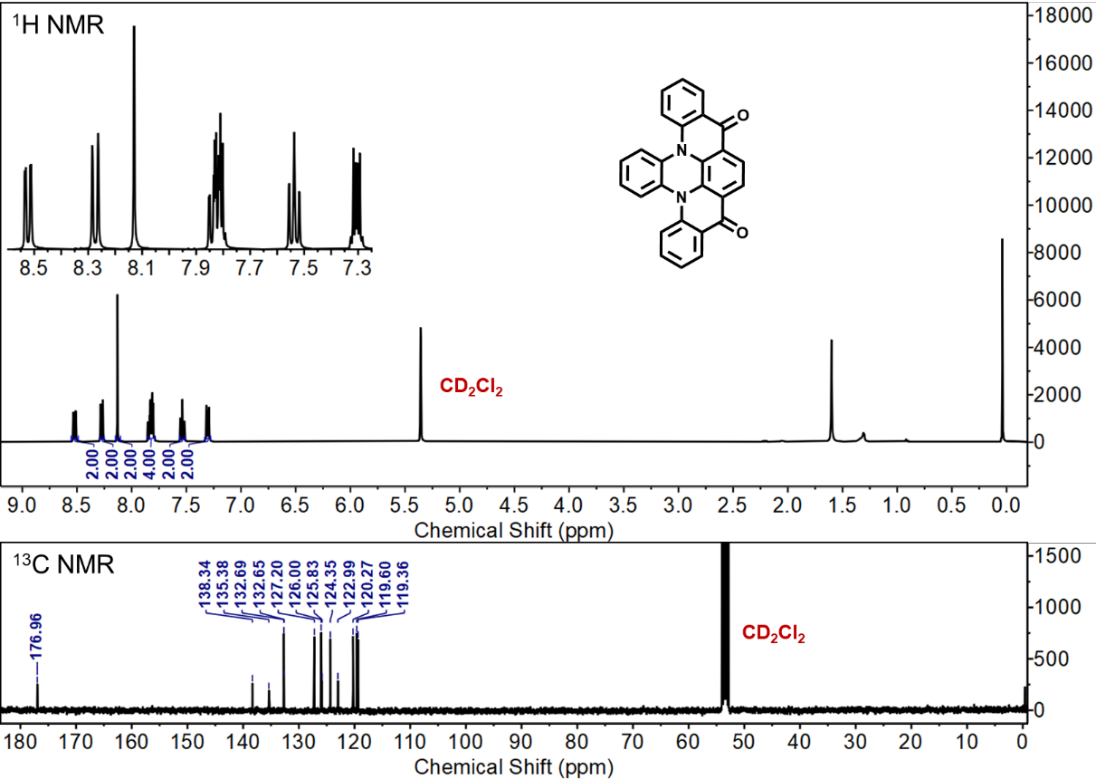


**Figure S2*.*** ^1^H and ^13^C NMR spectra of DNDK-2 in CD_2_Cl_2_ at room temperature.

**
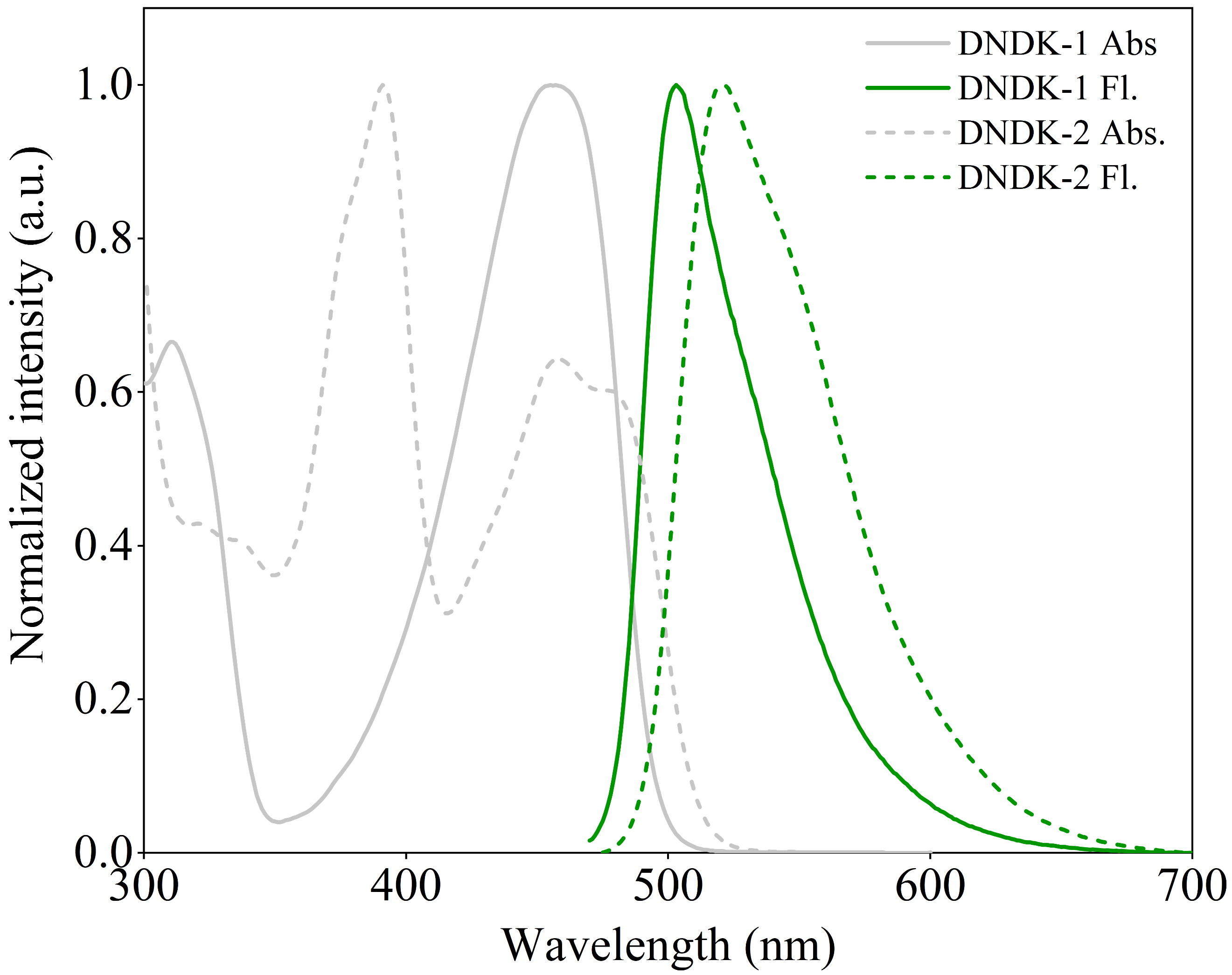
**

**Figure S3*.*** Ultraviolet–visible absorption and fluorescence spectra in dilute toluene at room temperature.


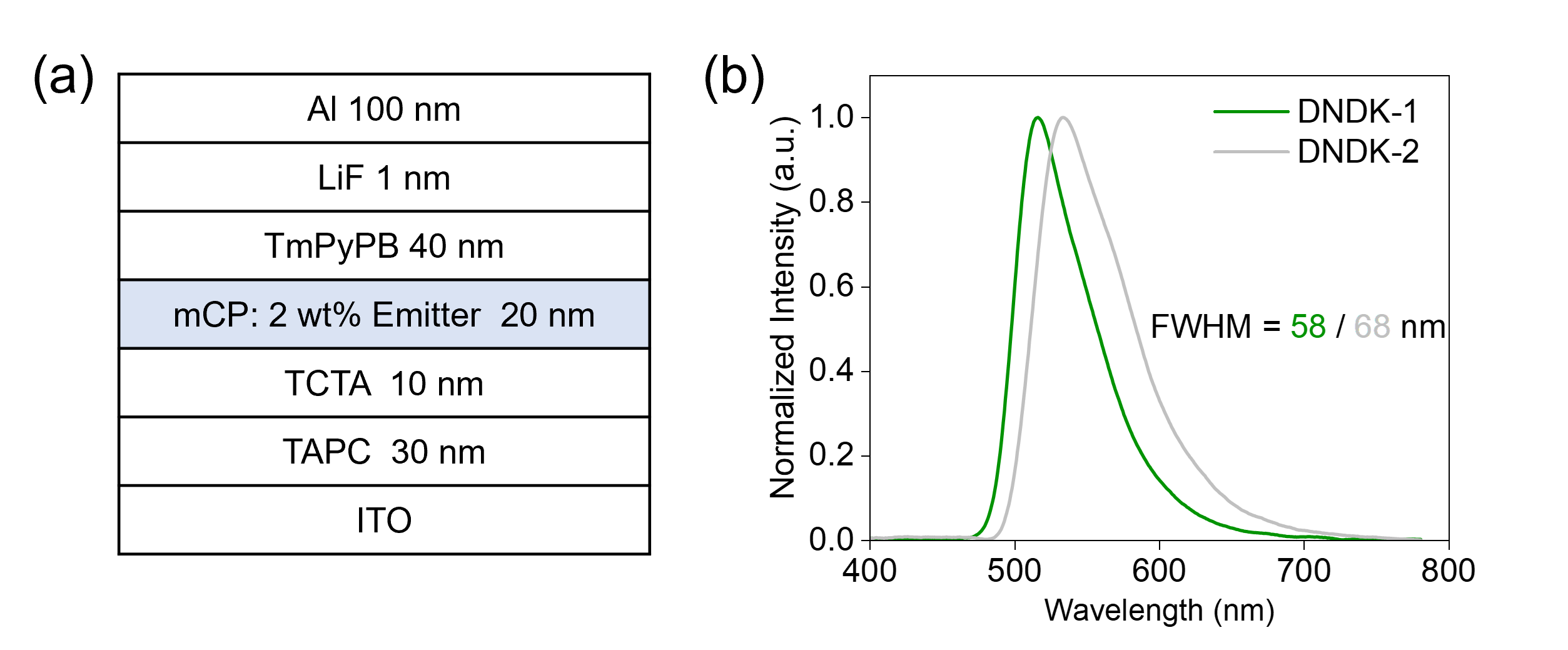


**Figure S4*.*** (a) Device structure and (b) Electroluminescence spectra at 1000 cd m^−2^ for OLEDs based on DNDK-1 and DNDK-2.

**Table S1**. The experimentally measured photophysical properties of DNDK-1 and DNDK-2.

| **Molecule** | $\boldsymbol{\lambda}_{\boldsymbol{abs}}$**^a^**  **[nm]** | $\boldsymbol{\lambda}_{\boldsymbol{em}}$**^a^**  **[nm]** | **FWHM^b^**  **[nm]** | $\boldsymbol{\lambda}_{\boldsymbol{fluo}}$**^c^**  **[nm]** | $\boldsymbol{\lambda}_{\boldsymbol{phos}}$**^c^**  **[nm]** | ***E*_S1_^d^**  **[eV]** | ***E*_T1_^d^**  **[eV]** | **Δ*E*_S1T1_^e^**  **[eV]** |
| --- | --- | --- | --- | --- | --- | --- | --- | --- |
| **DNDK-1** | 456 | 503 | 39 | 530 | 565 | 2.34 | 2.20 | 0.14 |
| **DNDK-2** | 479 | 520 | 66 | 537 | 636 | 2.31 | 1.95 | 0.36 |

^a^ Peak wavelength of absorption and fluorescence spectra in dilute toluene at room temperature.

^b^ Full-width at half-maximum.

^c^ Peak wavelength of fluorescence and phosphorescence spectra in doped film at 77K.

^d^ Determined from the peak wavelength of fluorescence and phosphorescence spectra in doped film at 77K.

^e^ Δ*E*_S1T1_; the singlet-triplet gap, i.e., *E*(S_1_)–*E*(T_1_).

**Table S2.** The excited state energies (in eV) of DNDK-1 and DNDK-2 calculated using various TD-DFT functionals.

| **Molecule** | **Excitation energy** | **ω-tuned ωB97XD** | **ω-default ωB97XD** | **M06-2X** | **B3LYP** | **CAM-B3LYP** |
| --- | --- | --- | --- | --- | --- | --- |
| **DNDK-1** | *E*(S_1_) | 3.18 | 3.54 | 3.47 | 2.87 | 3.50 |
|  | *E*(T_1_) | 2.50 | 2.78 | 2.81 | 2.34 | 2.72 |
|  | **Δ*E*_S1T1_** | 0.68 | 0.76 | 0.66 | 0.53 | 0.78 |
| **DNDK-2** | *E*(S_1_) | 2.96 | 3.31 | 3.26 | 2.72 | 3.27 |
|  | *E*(T_1_) | 2.12 | 2.46 | 2.47 | 2.07 | 2.40 |
|  | **Δ*E*_S1T1_** | 0.84 | 0.85 | 0.79 | 0.65 | 0.87 |

**
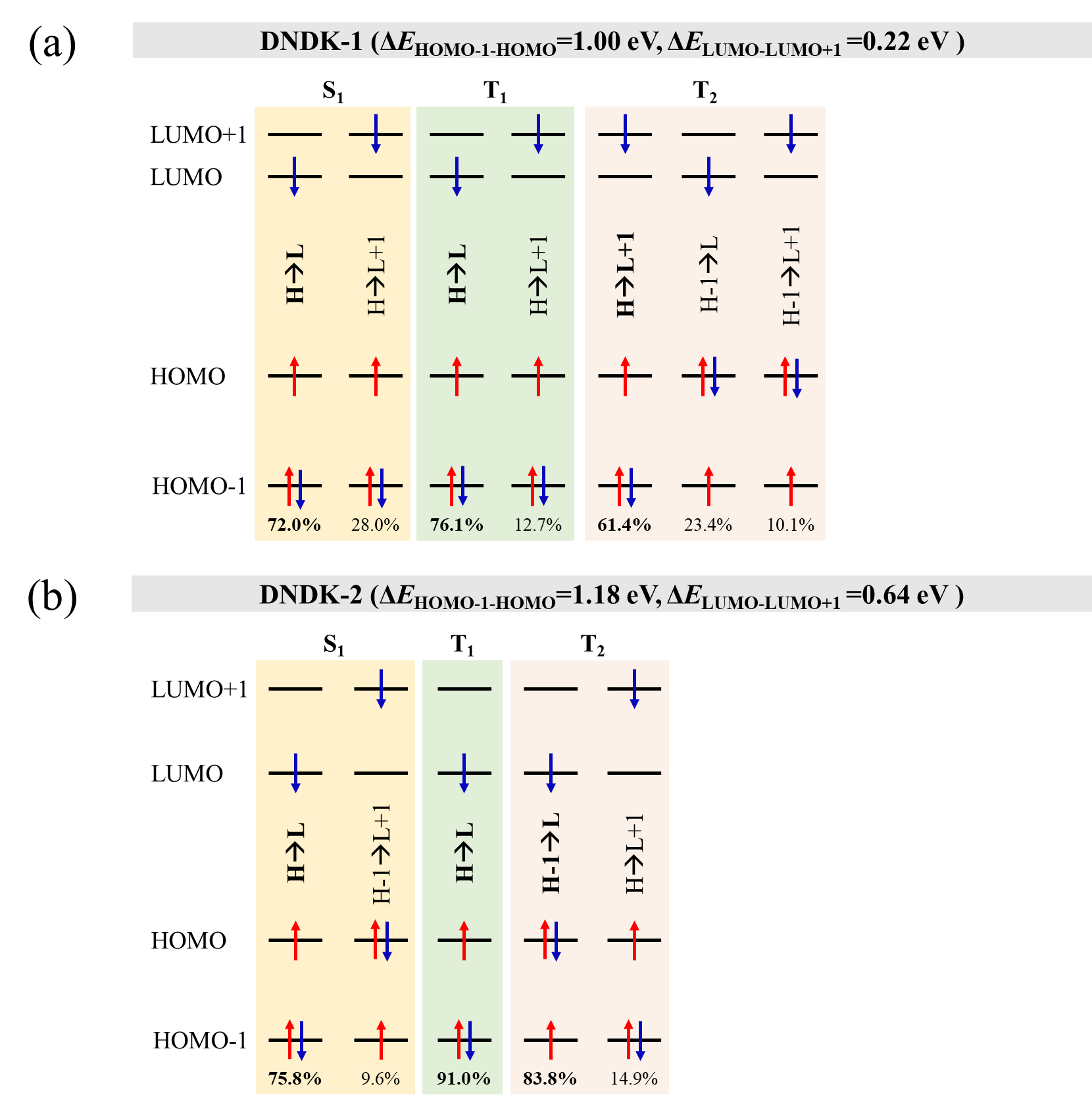
**

**Figure S5*.*** The major electronic transitions and their contributions (%) to the S_1_/T_1_/T_2_ state in (a) DNDK-1 and (b) DNDK-2. The symbols H-1, H, L and L+1 stand for the HOMO-1, HOMO, LUMO and LUMO+1, respectively. ΔE_HOMO-1-HOMO_ (ΔE_LUMO-LUMO+1_) denotes the energy gap between HOMO and HOMO-1 (LUMO+1 and LUMO). Red/blue arrows denote α/β spin of an electron.


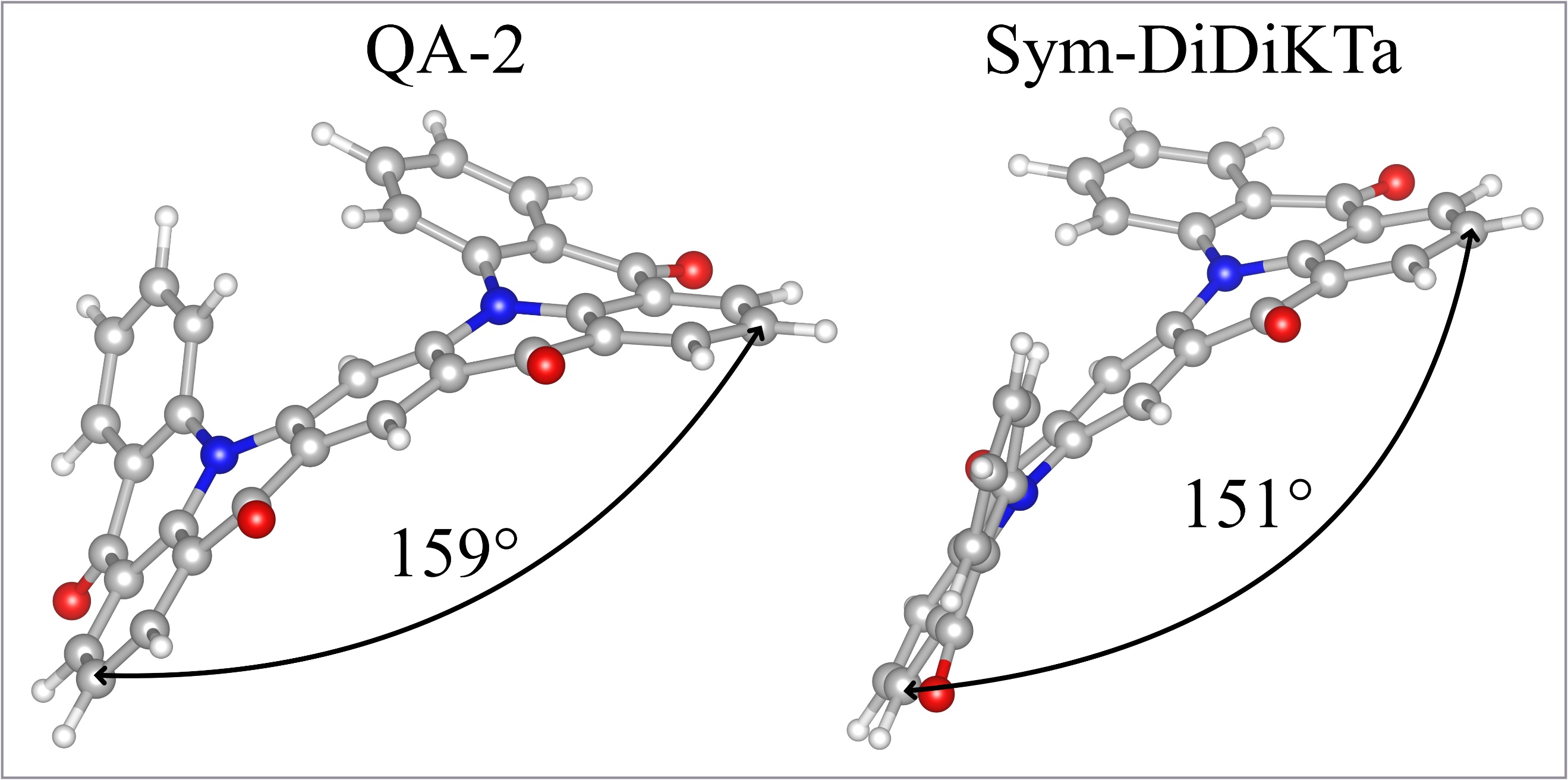


**Figure S6*.*** The distortion angles of the QA-2 and Sym-DiDiKTa backbones.


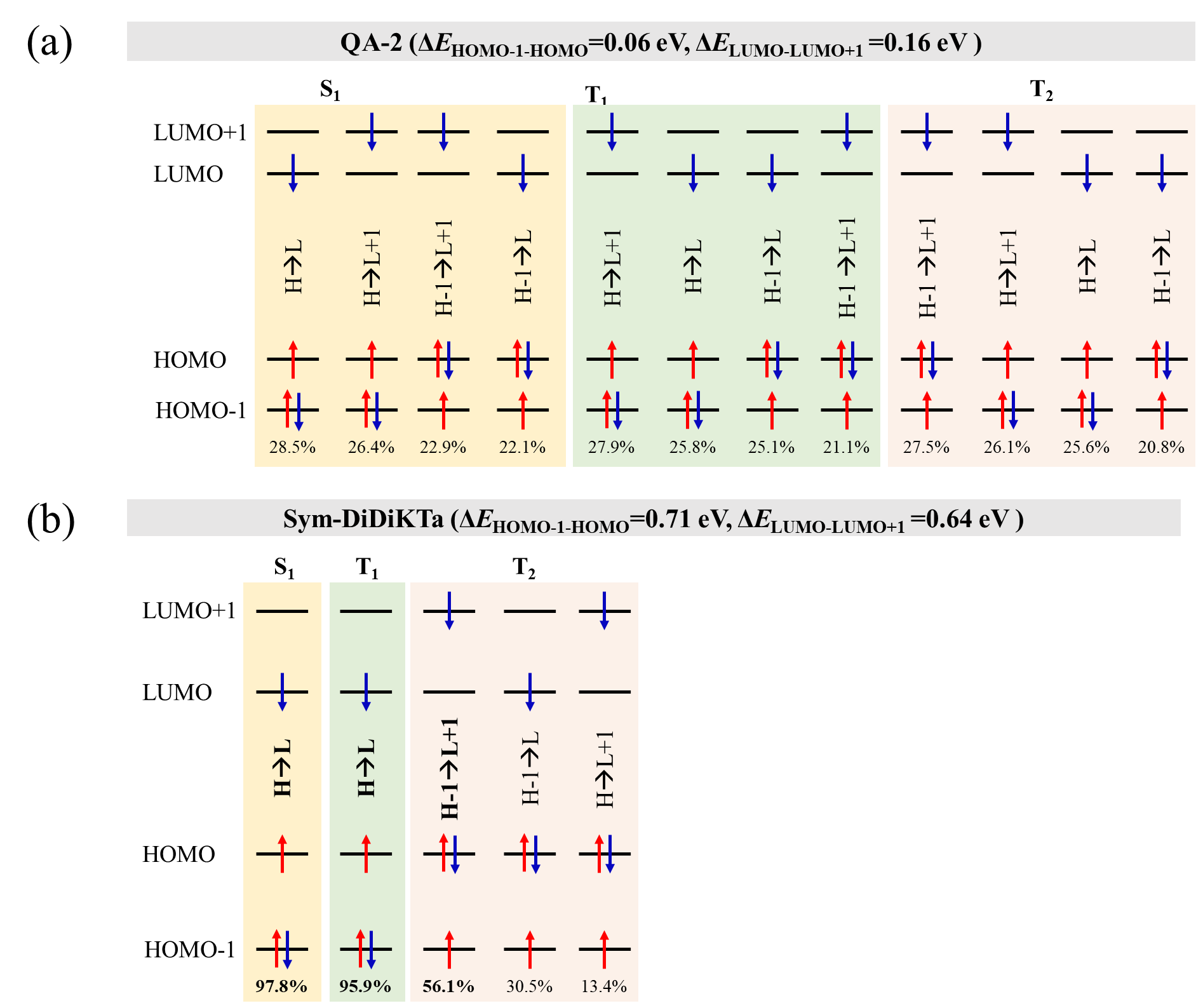


**Figure S7*.*** The major electronic transitions and their contributions (%) to the S_1_/T_1_/T_2_ state for (a) QA-2 and (b) Sym-DiDiKTa. The symbols H-1, H, L and L+1 stand for the HOMO-1, HOMO, LUMO and LUMO+1, respectively. ΔE_HOMO-1-HOMO_ (ΔE_LUMO-LUMO+1_) denotes the energy gap between HOMO and HOMO-1 (LUMO+1 and LUMO). Red/blue arrows denote α/β spin of an electron.


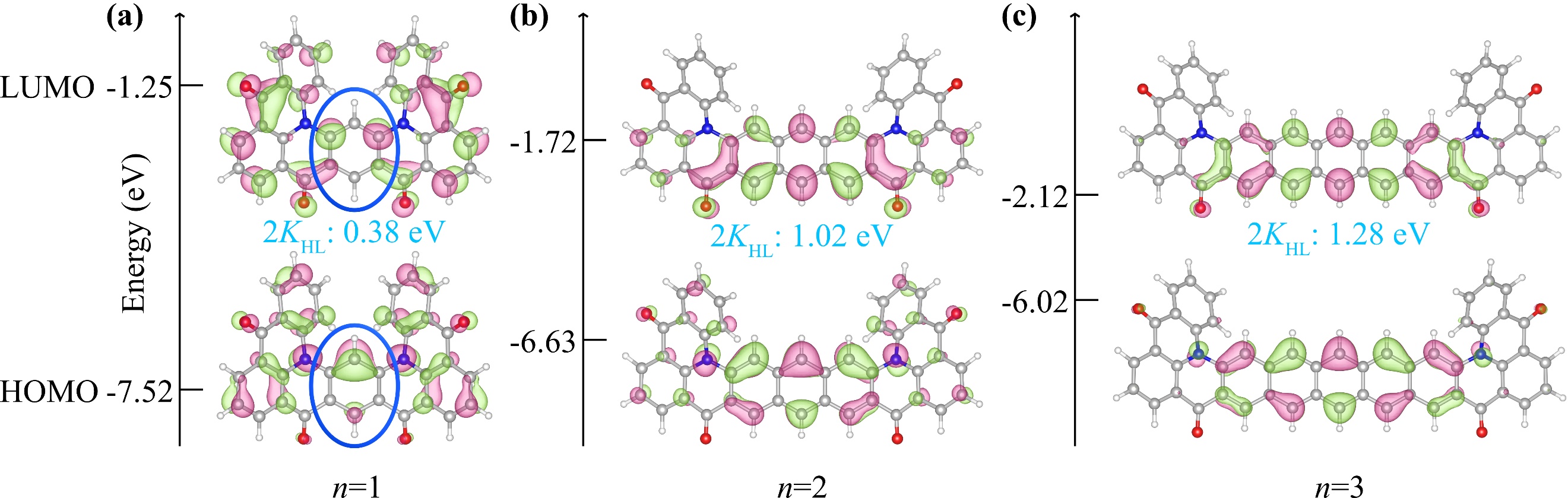


**Figure S8*.*** The change in frontier orbitals as a function of *n* (extent of π-conjugation) in QA-2*_n_* (see Figure 6 in main text). The region highlighted by blue eclipses depicts the hindrance of π-conjugation.

**References**

[1] M. J. Frisch, G. W. Trucks, H. B. Schlegel, G. E. Scuseria, M. A. Robb, J. R. Cheeseman, G. Scalmani, V. Barone, G. A. Petersson, H. Nakatsuji, X. Li, M. Caricato, A. V. Marenich, J. Bloino, B. G. Janesko, R. Gomperts, B. Mennucci, H. P. Hratchian, J. V. Ortiz, A. F. Izmaylov, J. L. Sonnenberg, Williams, F. Ding, F. Lipparini, F. Egidi, J. Goings, B. Peng, A. Petrone, T. Henderson, D. Ranasinghe, V. G. Zakrzewski, J. Gao, N. Rega, G. Zheng, W. Liang, M. Hada, M. Ehara, K. Toyota, R. Fukuda, J. Hasegawa, M. Ishida, T. Nakajima, Y. Honda, O. Kitao, H. Nakai, T. Vreven, K. Throssell, J. A. Montgomery Jr., J. E. Peralta, F. Ogliaro, M. J. Bearpark, J. J. Heyd, E. N. Brothers, K. N. Kudin, V. N. Staroverov, T. A. Keith, R. Kobayashi, J. Normand, K. Raghavachari, A. P. Rendell, J. C. Burant, S. S. Iyengar, J. Tomasi, M. Cossi, J. M. Millam, M. Klene, C. Adamo, R. Cammi, J. W. Ochterski, R. L. Martin, K. Morokuma, O. Farkas, J. B. Foresman, D. J. Fox, Wallingford, CT, **2016**.

[2] F. Neese, F. Wennmohs, U. Becker, C. Riplinger, *J. Chem. Phys.* **2020**, *152*.

[3] Q. Sun, T. C. Berkelbach, N. S. Blunt, G. H. Booth, S. Guo, Z. Li, J. Liu, J. D. McClain, E. R. Sayfutyarova, S. Sharma, S. Wouters, G. K.-L. Chan, *WIREs Comput. Mol. Sci.* **2018**, *8*, e1340.

[4] T. Lu, F. Chen, *J. Comput. Chem.* **2012**, *33*, 580-592.

[5] F. Weigend, R. Ahlrichs, *Phys. Chem. Chem. Phys.* **2005**, *7*, 3297-3305.
